# The complete mitochondrial genome sequences of two *Isospora* species (Eimeriidae, Eucoccidiorida, Coccidiasina, Apicomplexa) causing coccidiosis in superb glossy starlings, *Lamprotornis superbus* (Aves: Sturnidae)

**DOI:** 10.1080/23802359.2017.1407698

**Published:** 2017-11-27

**Authors:** Mian A. Hafeez, John R. Barta

**Affiliations:** a Department of Pathobiology, Ontario Veterinary College, University of Guelph, Guelph, ON, Canada;; b Department of Parasitology, University of Veterinary and Animal Sciences, Lahore, Pakistan

**Keywords:** Coccidia, fragmented rRNA, Isospora, parasitology, atoxoplasmosis

## Abstract

Complete mitochondrial genomes are reported for two *Isospora* species causing systemic coccidiosis in Superb Glossy Starlings (Aves: Sturnidae). The A/T rich (34.7% G/C) genomes were 6223 bp in length for *Isospora greineri* and 6217 bp for *Isospora superbusi*. Each encoded 3 protein-coding genes, (COI, COIII and CytB) plus 18 LSU and 14 SSU rDNA fragments. Arrangement of protein- and rRNA-coding regions was identical to known *Eimeria* sp. mt genomes; start codon usage was conventional. The mitochondrial genome structures of *Isospora* and *Eimeria* species are conserved and reflect the close phylogenetic association between these eimeriid genera of apicomplexan parasites.

Passeriformes (e.g. canaries, finches, sparrows, grosbeaks and starlings) and other birds are commonly parasitized by coccidia transmitted via the fecal-oral route (Box 1981; Levine 1982; Schrenzel et al. 2005). More than 90% of all the described coccidia infecting wild birds belong to the genus *Isospora* (see Pellérdy 1974). In passeriform birds, enteritis caused by *Isospora* spp. resembles coccidiosis in poultry and subclinical infections are common (Page and Haddad 1995). Systemic (extraintestinal) coccidiosis is caused by some *Isospora* species that migrate during merogony (Quiroga et al. 2000; Upton et al. 2001; Cushing et al. 2011; Hafeez et al. 2014). Host specificity of *Isospora* species is believed to be relatively narrow, perhaps at the host genus level, like many other eimeriid coccidia (Long 1982). Levine (1982) generalized that a particular coccidium is likely to be transmissible between host species within the same host genus but not to a different host genus, even if in the same family.

Specific identification based on microscopy is almost impossible because some *Isospora* species possess morphologically indistinguishable oocysts (Hafeez et al. 2014). Molecular data from avian *Isospora* species are scarce and, where available, are usually nuclear small subunit rDNA sequences (Carreno and Barta 1999). Schrenzel et al. (2005) also obtained short mt COI sequences in one of the first such uses of mt sequences in molecular studies of these coccidia. In the present study, the complete mitochondrial genome sequences are reported for two recently named *Isospora* spp. (see Hafeez et al. 2014) that caused systemic coccidiosis in captive Superb Glossy Starlings (*Lamprotornis superbus*; Aves: Sturnidae) held at the Toronto Zoo (43.819583, -79.184722).

DNA was extracted from oocysts and infected tissues containing numerous merozoites obtained during diagnostic necropsy (liver, spleen, lungs and intestine) as described (Hafeez et al. 2014) and the mt genome amplified using long-range PCR with coccidia-specific primers to obtain complete mt genomes from *Isospora greineri* (6223bp) and *Isospora superbusi* (6217bp) assembled using de novo sequence assembly within Geneious software (Version 6.1 and later, from http://www.geneious.com). The mitochondrial genomes of *I. greineri* (GenBank: KP108298.1; Canadian Museum of Nature hepatotype series CMNPA 2014-0002) and *I. superbusi* (GenBank: KT203396.1; Canadian Museum of Nature hepatotype series CMNPA 2014-0003) had three protein-coding genes (COI, COIII and CytB) as well as 18 LSU and 14 SSU rDNA fragments but no regions encoding tRNAs. The two *Isospora* spp. had 99.2% pairwise sequence identity with the majority of differences within the protein-coding or intergenic regions (Figure 1). Genome organization was identical to that observed in a variety of eimeriid mt genomes (Ogedengbe et al. 2014); start codon usage, start positions and TAA termination codons for COI, COIII and CytB were consistent with those documented previously for other coccidian mt genomes (Ogedengbe et al. 2013). Our amplification of overlapping PCR products covering the complete mt genome suggests that the *Isospora* spp. mt genome is physically either circular (like *Plasmodium* spp.; Feagin et al. 2012) or a concatemer of multiple genome copies; the latter form is probable because concatenated mt genomes are reported for closely related *Eimeria* spp. (see Hikosaka et al. 2010).

**Figure 1.**
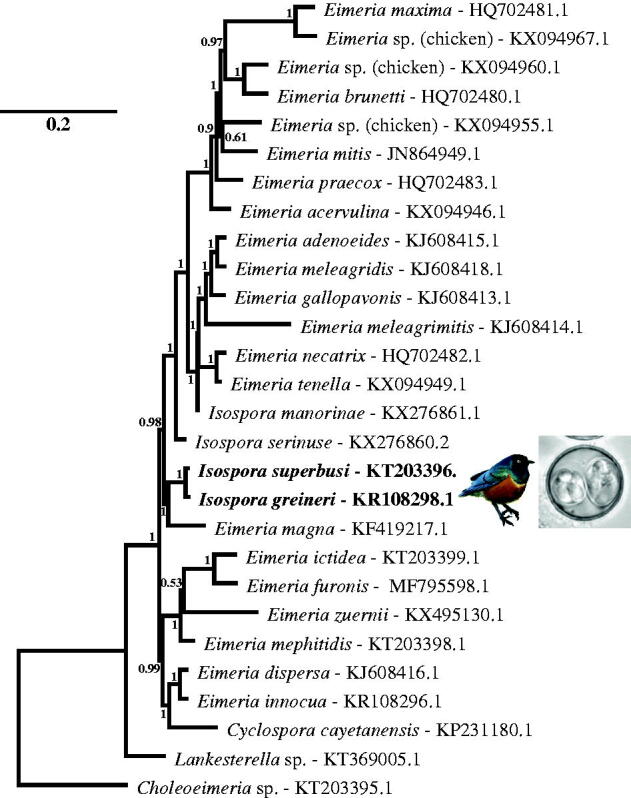
Phylogenetic tree based on the protein- and rRNA-coding regions of complete mitochondrial genome sequences from a variety of eimeriid coccidia demonstrates the close relationship between *Isospora superbusi* and *Isospora greineri*. The Bayesian analysis was performed on an alignment of 5394bp from each complete mitochondrial genome from the 3 CDS and 32 rDNA fragments; the dataset was partitioned so that the CDS were analyzed using a codon-based (mtmet translation) substitution model and the rDNA regions analyzed using a GTR + G + I substitution model. Scale bar indicates hypothesized evolutionary divergence and numbers at nodes indicate Bayesian posterior probabilities. Superb Glossy Starling image was modified from the original of Sumeet Moghe and used under license CC BY-SA 3.0.

## References

[CIT0001] Box ED. 1981 *Isospora* as an extraintestinal parasite of passeriforme birds. J Protozool. 28:244–246.

[CIT0002] Carreno RA, Barta JR. 1999 An eimeriid origin of isosporoid coccidia with Stieda bodies as shown by phylogenetic analysis of small subunit ribosomal RNA gene sequences. J Parasitol. 85:77–83. 10207368

[CIT0003] Cushing TL, Schat KA, States SL, Grodio JL, O’Connell PH, Buckles EL. 2011 Characterization of the host response in systemic isosporosis (atoxoplasmosis) in a colony of captive American goldfinches (*Spinus tristis*) and house sparrows (*Passer domesticus*). Vet Pathol. 48:985–992. 21311069 10.1177/0300985810391114

[CIT0004] Feagin JE, Harrell MI, Lee JC, Coe KJ, Sands BH, Cannone JJ, Tami G, et al 2012 The fragmented mitochondrial ribosomal RNAs of *Plasmodium falciparum*. PLoS One. 7:e38320. 22761677 10.1371/journal.pone.0038320 PMC3382252

[CIT0005] Hafeez MA, Stasiak I, Delnatte P, El-Sherry S, Smith DA, Barta JR. 2014 Description of two new *Isospora* species causing visceral coccidiosis in captive superb glossy starlings, *Lamprotornis superbus* (Aves: Sturnidae). Parasitol Res. 113:3287–3297. 24948107 10.1007/s00436-014-3992-8

[CIT0006] Hikosaka K, Watanabe Y, Tsuji N, Kita K, Kishine H, Arisue N, Palacpac NM, Kawazu S, Sawai H, Horii T, et al 2010 Divergence of the mitochondrial genome structure in the apicomplexan parasites, *Babesia* and *Theileria*. Mol Biol Evol. 27:1107–1116. 20034997 10.1093/molbev/msp320

[CIT0007] Levine ND. 1982 The genus *Atoxoplasma* (Protozoa, Apicomplexa). J Parasitol. 68:719–723. 7119994

[CIT0008] Long PL. 1982 The biology of the Coccidia. Baltimore (MD): University Park Press.

[CIT0009] Ogedengbe ME, Hafeez AM, Barta JR. 2013 Sequencing the complete mitochondrial genome of *Eimeria mitis* strain USDA50 (Apicomplexa: Eimeriidae) suggests conserved start positions for mtCOI- and mtCOIII-coding regions. Parasitol Res. 112:4129–4136. 24013344 10.1007/s00436-013-3604-z

[CIT0010] Ogedengbe ME, El-Sherry S, Whale J, Barta JR. 2014 Complete mitochondrial genome sequences from five *Eimeria* species (Apicomplexa; Coccidia; Eimeriidae) infecting domestic turkeys. Parasit Vectors. 7:335. 25034633 10.1186/1756-3305-7-335 PMC4223602

[CIT0011] Page CD, Haddad K. 1995 Coccidial infections in birds. J Exot Pet Med. 4:138–144.

[CIT0012] Pellérdy LP. 1974 Coccidia and coccidiosis. 2nd ed. Berlin: Verlag Paul Parey and Akademiai Kiady.

[CIT0013] Quiroga MI, Aleman N, Vazquez S, Nieto JM. 2000 Diagnosis of ‘atoxoplasmosis’ in a canary *Serinus canarius* by histopathologic and ultrastructural examination. Avian Dis. 44:465–469. 10879930

[CIT0014] Schrenzel MD, Maalouf GA, Gaffney PM, Tokarz D, Keener LL, McClure D, Griffey S, McAloose D, Rideout BA. 2005 Molecular characterization of isosporoid coccidia (*Isospora* and *Atoxoplasma* spp.) in passerine birds. J Parasitol. 91:635–647. 16108559 10.1645/GE-3310

[CIT0015] Upton SJ, Wilson SC, Norton TM, Greiner EC. 2001 A new species of *Isospora* Schneider, 1881 (Apicomplexa: Eimeriidae) from the Bali (Rothschild's) mynah *Leucopsar rothschildi* (Passeriformes: Sturnidae), and comments concerning the genera *Atoxoplasma* Garnham, 1950 and *Isospora*. Syst Parasitol. 48:47–53. 11213203 10.1023/a:1026520206525

